# Revisiting Respect for Persons in Genomic Research

**DOI:** 10.3390/genes5010001

**Published:** 2014-01-22

**Authors:** Debra J.H. Mathews, Leila Jamal

**Affiliations:** 1 Johns Hopkins Berman Institute of Bioethics, 1809 Ashland Avenue, Baltimore, MD 21205, USA; 2 Department of Health Policy and Management, Johns Hopkins Bloomberg School of Health, 615 North Wolfe St., Baltimore, MD 21205, USA; E-Mail: ljamal@jhsph.edu; 3 Division of Neurogenetics, Kennedy Krieger Institute, 801 N. Broadway, Rm. 564, Baltimore, MD 21205, USA

**Keywords:** respect for persons, genomics, genetics, informed consent

## Abstract

The risks and benefits of research using large databases of personal information are evolving in an era of ubiquitous, internet-based data exchange. In addition, information technology has facilitated a shift in the relationship between individuals and their personal data, enabling increased individual control over how (and how much) personal data are used in research, and by whom. This shift in control has created new opportunities to engage members of the public as partners in the research enterprise on more equal and transparent terms. Here, we consider how some of the technological advances driving and paralleling developments in genomics can also be used to supplement the practice of informed consent with other strategies to ensure that the research process as a whole honors the notion of respect for persons upon which human research subjects protections are premised. Further, we suggest that technological advances can help the research enterprise achieve a more thoroughgoing respect for persons than was possible when current policies governing human subject research were developed. Questions remain about the best way to revise policy to accommodate these changes.

## 1. Introduction

The risks and benefits of research using large databases of personal information are evolving in an era of ubiquitous, internet-based data exchange. Here, we consider some of the technological advances driving and paralleling developments in genomics, and how they can be used to supplement the practice of informed consent to ensure that the research process as a whole honors the notion of respect for persons upon which human research subjects protections are premised.

The cost of next-generation sequencing has declined precipitously in recent years, increasing the potential of genomic research to expand knowledge of human biology and disease [1]. To render human genome data meaningful for individuals, investigators must collect and analyze information contributed by many individuals from diverse populations over long periods of time. To build large datasets, people are asked to donate biospecimens and personal data, including genomic data, to repositories of de-identified tissue and data used by many researchers [2]. Indeed, in an effort to harness the scientific potential of such large datasets, many of the world’s leading research institutions recently announced ambitious plans to build a global, interoperable framework for sharing genomic and other research data more broadly in the future [3], and the NIH is currently developing a revised data-sharing policy [4]. As this new era of genomic research progresses, it is critical that we attend not only to the benefits that such broad sharing will have for science and medicine, but also to the proportionality of risks and benefits borne by contributors to biorepositories and genome databases.

The structures and norms guiding the development and use of such repositories were established at a time when the re-identification of individual data contributors was thought to be unlikely, and the anonymization of personal data was a reasonable strategy for mitigating risks to research subjects from loss of confidentiality and subsequent discrimination. As we have learned over the past five years, it is no longer possible to credibly guarantee that anonymized or de-identified samples and data will remain de-identified in large data repositories [5,6,7]. The increased technical capacity to reidentify individuals in databases can be addressed in a number of ways: (1) we can clamp down on sharing; (2) we can merely be transparent about the risks during the informed consent process and allow those individuals willing to assume the risks to do so [8]; or (3) we can shift our attention to increasing penalties for re-identification and misuse of identifiable data [9]. Limiting use would be an unfortunate and ill-considered outcome, reducing research and medical benefits to society and foiling the intentions of many individual contributors who are, after all, providing samples and data to further science and clinical innovation. Transparency and penalties for misuse may be necessary to address the increased risk of re-identification, but they are not sufficient. Here, we suggest that, where technological capacity exists, technological advances can help the research enterprise achieve a more thoroughgoing respect for persons than was possible when current policies governing human subject research were developed. Further, by restricting access to data and failing to recognize that some individuals may exercise their autonomy by enabling use of their genomic and personal data, researchers and regulators hobble science and fail to truly honor the notion of respect for persons that underlies the entire enterprise. That said, questions remain about the best way to revise policy to accommodate the changed landscape.

## 2. Background

Concerns about the ethical use of human genomic and other personal data in prospective cohort studies are longstanding [10]. However, the increased use of next-generation sequencing in research reanimates three challenges on an unprecedented scale. First, next-generation sequencing can generate data from every known disease-associated gene or DNA sample. As more is learned about the contribution of genomic factors to disease risk, an individual genome sequence will acquire new meaning to the person from whom it originated and will contribute to the interpretation of others’ genomes.

Second, next-generation sequencing has co-evolved with powerful computing infrastructures for analyzing and exchanging enormous volumes of personal data. To facilitate the efficient use of resources, there has been a growing tendency to establish large databases and open-access policies to store and share human genomic and other research data. This trend favors the “emergence” of many hypotheses from large datasets long after a participant’s initial informed consent to research, and facilitates the re-use and combining of datasets by multiple researchers. As a result, secondary and tertiary data users may be far removed from the original context in which research data were obtained, blurring the lines of accountability for responsible data use.

Third, it has become easier to re-identify individual contributors to databases based on publicly-available internet data, as the latter has grown more abundant [5,6,7]. Consequently, the privacy risks associated with contributing biospecimens and genomic data to research must now be assessed broadly, rather than in relation to the activities of any one project.

A current challenge facing policymakers is to develop standards for using not only archived tissues samples and data, but also newly generated genomic information in research to benefit society while respecting heterogeneous beliefs about privacy [11,12,13,14] and while safeguarding research participants from uncertain risks. This dilemma is often framed as a tension between serving individual autonomy interests by keeping data confidential on the one hand, and advancing public beneficence by sharing data liberally on the other. However, this polarized view may be oversimplified. Internet users have increasingly come to use social media—blogs, Facebook, Twitter, wikis, forums—to become content creators and sharers in their own right. While norms are still evolving, information technology (IT) has facilitated a shift in the relationship between individuals and their personal data, enabling increased individual control over how (and how much) personal data are used in research, and by whom. This shift in control has created new opportunities to engage members of the public as partners in the research enterprise on more equal and transparent terms. Conceptions of privacy—including what should remain private and what privacy means in various online spaces—and risks of breaching confidentiality are changing even as genomic data are accumulating rapidly.

## 3. The Rationale for Informed Consent

An ethical duty to secure the autonomous and voluntary informed consent of human research subjects emerged in response to specific and grave concerns—about physical harm, discrimination, stigma—that arose from inhumane and coercive research practices in the U.S., Europe and elsewhere during the 20th century [15,16]. Today, to uphold the bioethical principle of respect for persons, the United States Federal Policy for the Protection of Human Subjects (“The Common Rule”) requires investigators to obtain informed consent from prospective research subjects before collecting or using their individually identifiable biological materials or data in research studies [17]. The doctrine of informed consent was conceived to ensure respect for persons as autonomous agents in clinical care and research. Motivated to prevent further unethical research practices, the U.S. National Research Act of 1974 both mandated Institutional Review Board (IRB) review for research and convened a National Commission for the Protection of Human Subjects of Biomedical and Behavioral Research, which produced The Belmont Report, the foundation of much of the Common Rule. 

The Belmont Report identifies three ethical principles: respect for persons, beneficence, and justice, which are paired with three corresponding means of translating principle into action: informed consent, assessing risks and benefits, and fair selection of subjects. The original Belmont concept of “autonomy” embedded in respect for persons is elaborated as follows:

An autonomous person is an individual capable of deliberation about personal goals and of acting under the direction of such deliberation. To respect autonomy is to give weight to an autonomous person’s considered opinions and choices while refraining from obstructing their actions unless they are clearly detrimental to others. To show lack of respect for an autonomous agent is to repudiate that person’s considered judgments or to withhold information necessary to make a considered judgment, when there are no compelling reasons to do so [18] [underlining added].

The Belmont Report formed the basis of the first formal research regulations adopted by the Department of Health and Human Services (HHS) in 1981, only slightly modified in the currently prevailing Common Rule.

## 4. The Changing Research Landscape

It is widely agreed that since the adoption of the Common Rule, the advent of genomic research has changed the research landscape, as have its risks and benefits, as a result of technological advances that make it cheaper and easier to generate, analyze, and share large volumes of data [19,20]. Just as significant, many technological advances in the same period have diversified the tools available to mitigate or offset the risks facing contributors to genomic research.

### 4.1. The Shifting Relationship between Identifiability and Ethics Review

Historically, the risks of genetic and genomic research have been mitigated by nondisclosure (e.g., of non-paternity), and sample and data anonymization or de-identification. Stripping identifiers or severing links between tissues and tissue donors were, justifiably, seen as effective measures to mitigate risks to individuals’ privacy interests, by restricting access to their personal information. Yet privacy is a complex, variably defined concept encompassing a plurality of related issues; informational secrecy is merely one of its dimensions. Further, the practice of respecting privacy by restricting access to individual information undermines the pursuit of public benefit through aggregation of large amounts of personal data in research databases, and may not actually align with research subjects’ values [21,22].

The concerns addressed by restricting access to personal information include threats to valued social and economic opportunities as a result of privacy breaches and threats to individual autonomy, including risk of social stigma and unwanted scrutiny, making it harder to exercise basic liberties in the course of daily life [23]. Further, some individuals simply do not want others (e.g., researchers) to know information about them that they do not know themselves, or that they do not wish to know about themselves.

The moral case for gaining access to personal information also varies. In science, the argument is often made that such access will advance scientific knowledge, leading to improved healthcare and other societal benefits [24,25]. Justifying the use of personal information to achieve ends like these is difficult when the contribution of individual information to these outcomes is unclear, and even harder when not all parties involved are in agreement about the desirability of the ends. The various interests protected and hindered by confidentiality provisions make it impossible to arrive at a consensus risk-benefit profile for a pool of research subjects that can be assessed each time personal information is transferred from one holder to another.

Given the choice, some individuals might decline to make their personally identifiable health information available to researchers; others might elect to share their data to enable scientists to develop new treatments, to help advance biomedical science, or to forge connections to other individuals with common diagnoses or health concerns; still others might choose to share with academic but not commercial researchers, or with breast cancer researchers, but not those who study psychiatric disease. Whether a person is motivated to enroll in research by personal history of illness, intellectual curiosity, or feelings of altruism or social responsibility, the tradeoffs involved in contributing personal information to a biorepository are dynamic and variable over time, and contributors’ values and goals are diverse. Current policy that uniformly restricts access to data as a form of privacy protection both fails to respect those participants who would wish to have and share their data freely and limits the potential benefits to science and society that may accrue from the use of those data.

In recent years, it has become increasingly possible to re-identify individual data contributors to large electronic datasets [5,6,7]. This is significant because under the regulatory status quo, full ethics review is primarily reserved for projects using personal data considered “identifiable” under the Common Rule, meaning that the identity of the subject can be “readily ascertained” by the investigator from the information. Informed consent is not typically sought from individuals before their “de-identified” data are used in research. In human genomics, this policy is problematic due to the inherent identifiability of human sequence data and the need sometimes to interpret these data in the context of detailed phenotypic information.

The prevailing notion that investigators can balance the risk-benefit profile of genomic research by divorcing data from individual identifiers is also problematic because de-identification may actually impoverish the quality of research data to an extent that undermines scientific progress. De-identification might also preclude the return of individual research results to participants in instances when such results have implications for their well-being. Further, de-identification denies participants the opportunity to exercise their autonomy by managing the use of their data over time, as their circumstances and views change. From an individual’s perspective, the foreclosure of these benefits and limitations on their autonomy might actually worsen the risk-benefit profile of participating in research. 

### 4.2. Growth of Online Data-Sharing

Simultaneous with the emergence of next-gen sequencing technologies, there has been a profound shift in the nature of online information sharing in the course of daily life. Today’s Internet contains vast quantities of user-volunteered, identifiable data disclosed for purposes as varied as commercial exchange, social networking, recreational gaming, and health support and promotion. Facebook, Pinterest, patient discussion boards, posted Fitbit reports and myriad other forms of Internet sharing have changed what, how and with whom we share. In many online health-related communities, members develop and test their own hypotheses, assuming roles typically reserved for “experts”, and operating outside traditional human subjects protections frameworks (see Section 5.4 below). Further, some have begun to advocate not for the ability to keep one’s data private, but rather for the ability to have and to share one’s data freely [26]. Such calls for the freedom to share reflect the oft-ignored feature of autonomy as defined in the Belmont Report, respect for individuals’ ability to pursue their interests so long as they do not harm others (see underling above).

Norms of information exchange are also changing. When investigators and institutions are trusted, research participants tend not to mind contributing identifiable data to multiple research projects provided that they are kept informed about the nature of the research to which they are contributing [27,28]. Furthermore, several studies have shown that individual concerns about privacy are highly variable and seem to be affected by the tradeoffs that individuals make among three considerations: their privacy concerns, their perceptions of the utility of study participation, and the degree of reciprocity they perceive from investigators using their data [29,30].

Taken together—the limitations of informed consent, the growing ease of re-identifying donors and the value of donor-associated data, the proliferation of new IT platforms, and evidence for a so-called “privacy-utility tradeoff” made by research participants—these new realities suggest it is time to revise how we configure an ethical relationship between donors and users of genomic research data. If we wish to uphold the notion of respect for persons on which we base human research subject protections, we must both “give weight to an autonomous person’s considered opinions and choices” and refrain “from obstructing their actions unless they are clearly detrimental to others.” Limiting autonomy by restricting individuals’ access to and sharing of their own data, or ability to modify their preferences regarding data use over time fails to uphold the second requirement of respect for persons.

## 5. Application of IT to both Research and Research Subject Protections

The importance of trust and reciprocity to research participation suggests that revising the relationship between donors and users toward a more collaborative model might also encourage and support participation in genomic research, to the potential benefit of both parties and society as a whole. Many argue that research subjects must become more active partners in the research process itself: true participants, rather than mere subjects [10,11,12]. To realize this aim, and achieve the hoped for trust and reciprocity, new digital systems for collecting and curating research data (including genomic data) have been developed by innovators in both the for-profit and non-profit sectors. Below, we describe a heterogeneous group of evolving new approaches to collecting and using biospecimens and genomic data in research. Given their novelty and continuing evolution, it is not our aim to classify them prematurely or draw a false equivalence among them. Our goals are to draw attention to the innovative ways these approaches re-imagine the relationship between research participants and researchers, and to highlight some of the empirical questions that must be addressed, as we attempt to evaluate the ethical implications of the new research models.

### 5.1. The Personal Genome Project and Open Consent

The Harvard-based Personal Genome Project (PGP) [31] has abandoned the notion that de-identification of genomic research data and samples is plausible or even desirable, privileging the values of “veracity” and reciprocity in the conduct of research [32]. The PGP is a longitudinal genome research study enrolling participants through a detailed, web-based informed consent process (including a mandatory genetics exam) that secures “open consent” from participants for ongoing research use of their individual genomic and phenotypic data. PGP participants are free to upload as little or as much personal information as they wish to their online PGP profiles, within its defined parameters. Although these profiles do not display names, the PGP makes no promises that data contributed to the project will remain de-identified or anonymous. In return for assuming the risks of re-identification, the PGP offers participants individual research data and hosts an annual research meeting to which participants are invited, demonstrating the PGP’s belief that reciprocity may play an important role in earning and securing the trust of their study participants.

### 5.2. Portable Legal Consent

The Portable Legal Consent (PLC), developed by the Consent to Research project, is designed to address the challenges of broad data sharing. The PLC gives participants who wish to donate data to research the opportunity to attach a single research consent to their health and genetic data, which they then upload to a secure website. These data can then be used for research purposes by any researcher who agrees to specific terms of data use including: an intent to publish research results in an open-access forum, a promise not to attempt to re-identify individual research participants, and a promise not to distribute data among third parties who do not agree to the PLC conditions. While participants may withdraw their data from the database at any time, they are clearly advised that once data are uploaded, it may not be possible to remove them from all sources (for example, from researchers who have already downloaded, shared, or used the data).

### 5.3. Registry for All Disease (“Reg4All”)

In 2012, the umbrella disease advocacy organization Genetic Alliance created Reg4All [33] to collect information relevant to many health conditions. Using a “dynamic consent” platform, Reg4All participants select fine-grained consent rules to determine how their personal data are viewed, by whom, and for what purposes. The system’s privacy settings include “deny the use of my data in any form for any purpose”; “allow discovery and retrieval of all of my data in the registry”, and “make my data available to ONLY this research project”. Preferences also allow varying degrees of contact between registry participants and investigators interested in using their data. Participants may make their data available to specific clinical trials and research studies, or they may allow their data to be used openly by all. For each decision about data use, a participant may choose to give consent, deny consent, or postpone the decision until later. A participant may choose to enter their preferences once and retain them, or they may choose to change their choices at a later date. The overall vision of Reg4All is to re-imagine the researcher-participant relationship as a reciprocal collaboration over time.

### 5.4. “Apomediated”, Peer-Produced Research

The term “apomediation” describes the relatively non-hierarchical nature of information-sharing in some research communities [34,35]. Apomediated initiatives create virtual spaces in which individuals are encouraged to propose and carry out their own research studies using self-reported data. Examples include PatientsLikeMe (PLM), which provides self-tracking and social networking tools to its over 220,000 users in exchange for permission to share their data with researchers listed on the PLM website. Since 2012, PLM’s peer-reviewed publications have covered measures of functional disability in multiple sclerosis, epilepsy care quality, and Parkinson’s disease progression [36,37,38]. Other initiatives include DIYGenomics, which has hosted a crowd-sourced study of the relationship between polymorphisms in the Methylenetetrahydrofolate reductase (MTHFR) gene, homocysteine levels, and vitamin B deficiency, and Genomera, which in beta version allows members of online communities to initiate studies related to nutrition, sleep patterns, exercise, and genome variation [39]. 

## 6. Open Questions

The ability of IT and social media to change how genomic and other health data are shared and interpreted has generated excitement among health-oriented constituencies. Advocacy organizations have embraced social media’s role in helping patients become more engaged in their own healthcare and in research [40,41,42]. That said, using social media to share personal information raises its own ethical issues, and robust, longitudinal studies examining the effectiveness or safety of using social media to manage health information are needed. Some question whether existing initiatives are as “participant-centric” as they claim, given that commercial incentives may generate conflicts of interest in some cases [43]. One obvious concern is that personal information may be acquired surreptitiously or abused [44]. Another concern is that “gamified” survey data may not always be contributed voluntarily by users, given the compulsive nature of some forms of internet gaming [45]. Yet other concerns focus on financial motivations of the entities controlling the data—will participant and researcher incentives always stay in alignment [43]?

Thus far, we have few data on basic questions about these new models for doing research, such as: do granular data-sharing choices unduly hinder or bias the collection of research data? Who, if anyone, is alienated or excluded by systems like those we have described above? It is important to acknowledge that many participants in genomics research will not have ready access to or experience with the kinds of technologies we discuss here—will variation in access to technology lead to or exacerbate existing disparities between different research populations? Which, and how many, data-sharing options are necessary to secure autonomous and respectful research participation? What happens when study participants assume roles traditionally held by researchers?

Interactive websites have been demonstrated to be effective at educating the public about genomics, and individual data-sharing attitudes have been found to be highly nuanced and variable. We believe that the approaches highlighted above are promising strategies for managing many of the challenges of modern genomic research, while fostering autonomy. However, to realize their full potential, they must be developed in parallel with empirical studies of their benefits and harms, both intended and unintended.

## 7. Conclusions

Current informed consent practices are unequal to the task of upholding authentic respect for persons in contemporary genomic research. New models that take advantage of advances in both genomic research and IT promise to address this shortfall, but require further study of their associated benefits and harms. Careful study will be necessary to guide the evolution of these new models, and to ensure that research both adequately balances protections and benefits against the burdens and uncertainties borne by participants in genomic studies, and does not unnecessarily limit participants’ actions.

Prior work in bioethics has addressed privacy concerns narrowly, by focusing on privacy as a strict function of identifiability or a form of informational secrecy [46,47,48]. This focus misses other broad interests individuals may have in sharing their own health and genomic data and information. The conception of privacy as informational secrecy lends itself to a view of genomic information-sharing as a false dichotomy, in which information is either wholly private or wholly public. By restricting access to data and failing to recognize that some individuals may exercise their autonomy by enabling use of their genomic and personal data, researchers and regulators hobble science and fail to truly honor the notion of respect for persons that underlies the entire enterprise.

The scientific, bioethics, and research oversight communities frequently frame the debate as privacy *versus* public beneficence and equate respect for persons with informed consent. Such norms and practices impede meaningful reform of human subjects protections. Further, we lack the empirical evidence necessary to evaluate emerging models of engaging with research subjects and participants that more fully embody the original concept of respect for persons. The research enterprise as a whole must accommodate the cultural shift that is taking place in the relationship between individuals and their health information. Appreciating and understanding this transformation will be an indispensible step in adapting ethical guidelines to the realities of modern information use and patients who want and expect to be true participants in research.

## References

[1] Pasche B., Absher D. (2011). Whole-genome sequencing: A step closer to personalized medicine. JAMA.

[2] Greenbaum D., Sboner A., Mu X.J., Gerstein M. (2011). Genomics and privacy: Implications of the new reality of closed data for the field. PLoS Comput. Biol..

[3] Hayden E.C. (2013). Geneticists push for global data-sharing. Nature.

[4] Draft NIH Genomic Data Sharing Policy Request for Public Comments; 2013. http://www.bioethics.net/2013/10/nih-requests-comment-on-genomic-data-sharing-policy-draft/.

[5] Gymrek M., McGuire A.L., Golan D., Halperin E., Erlich Y. (2013). Identifying personal genomes by surname inference. Science.

[6] Homer N., Szelinger S., Redman M., Duggan D., Tembe W., Muehling J., Pearson J.V., Stephan D.A., Nelson S.F., Craig D.W. (2008). Resolving individuals contributing trace amounts of DNA to highly complex mixtures using high-density SNP genotyping microarrays. PLoS Genet..

[7] Sweeney L.A.A., Winn J. (2013). Identifying Participants in the Personal Genome Project by Name.

[8] Lunshof J.E., Chadwick R., Vorhaus D.B., Church G.M. (2008). From genetic privacy to open consent. Nat. Rev. Genet..

[9] Bambauer J.Y. (2011). Tragedy of the data commons. Harv. J. Law Technol..

[10] Geller L.N., Alper J.S., Billings P.R., Barash C.I., Beckwith J., Natowicz M.R. (1996). Individual, family, and societal dimensions of genetic discrimination: A case study analysis. Sci. Eng. Ethics.

[11] McGuire A.L., Caulfield T., Cho M.K. (2008). Research ethics and the challenge of whole-genome sequencing. Nat. Rev. Genet..

[12] Kaye J. (2012). The tension between data sharing and the protection of privacy in genomics research. Annu. Rev. Genomics Hum. Genet..

[13] (2012). Privacy and Progress in Whole Genome Sequencing.

[14] Knoppers B.M., Dove E.S., Litton J.E., Nietfeld J.J. (2012). Questioning the limits of genomic privacy. Am. J. Hum. Genet..

[15] Rice T.W. (2008). The historical, ethical, and legal background of human-subjects research. Respir. Care.

[16] Menikoff J. (2013). Research involving human subjects: Ethical and regulatory issues. Handb. Clin. Neurol..

[17] (2008). Code of Federal Regulations Title 45, Section 46. Federal Policy for the Protection of Human Subjects.

[18] The National Commission for the Protection of Human Subjects of Biomedical and Behavioral Research (1979). The Belmont Report: Ethical Principles and Guidelines for the Protection of Human Subjects of Research.

[19] Meslin E.M., Cho M.K. (2010). Research ethics in the era of personalized medicine: Updating science’s contract with society. Public Health Genomics.

[20] Bunnik E.M., de Jong A., Nijsingh N., de Wert G.M. (2013). The new genetics and informed consent: Differentiating choice to preserve autonomy. Bioethics.

[21] Mello M.M., Wolf L.E. (2010). The havasupai Indian tribe case—Lessons for research involving stored biologic samples. NEJM.

[22] McGuire A.L., Oliver J.M., Slashinski M.J., Graves J.L., Wang T., Kelly P.A., Fisher W., Lau C.C., Goss J., Okcu M. (2011). To share or not to share: A randomized trial of consent for data sharing in genome research. Genet. Med..

[23] Powers M., Rothstein M.A. (1997). Justice and Genetics: Privacy protection and the Moral Basis of Public Policy. Genetic Secrets: Protecting Privacy and Confidentiality in the Genetic Era.

[24] (2013). Sharing Clinical Research Data: Workshop Summary.

[25] Terry S.F., Shelton R., Biggers G., Baker D., Edwards K. (2013). The haystack is made of needles. Genet. Test. Mol. Biomark..

[26] Terry S.F., Terry P.F. (2011). Power to the people: Participant ownership of clinical trial data. Sci. Transl. Med..

[27] Ludman E.J., Fullerton S.M., Spangler L., Trinidad S.B., Fujii M.M., Jarvik G.P., Larson E.B., Burke W. (2010). Glad you asked: Participants’ opinions of re-consent for dbGaP data submission. JERHRE.

[28] Lemke A.A., Wolf W.A., Hebert-Beirne J., Smith M.E. (2010). Public and biobank participant attitudes toward genetic research participation and data sharing. Public Health Genomics.

[29] Oliver J.M., Slashinski M.J., Wang T., Kelly P.A., Hilsenbeck S.G., McGuire A.L. (2012). Balancing the risks and benefits of genomic data sharing: Genome research participants’ perspectives. Public Health Genomics.

[30] Hobbs A., Starkbaum J., Gottweis U., Wichmann H.E., Gottweis H. (2012). The privacy-reciprocity connection in biobanking: Comparing german with UK Strategies. Public Health Genomics.

[31] Personal Genome Project. http://www.personalgenomes.org/.

[32] Angrist M. (2009). Eyes wide open: The personal genome project, citizen science and veracity in informed consent. Per. Med..

[33] Reg4All. https://reg4all.org/.

[34] Eysenbach G. (2008). Medicine 2.0: Social networking, collaboration, participation, apomediation, and openness. J. Med. Int. Res..

[35] O’Connor D. (2013). The apomediated world: Regulating research when social media has changed research. J. Law Med. Ethics.

[36] Wicks P., Fountain N.B. (2012). Patient assessment of physician performance of epilepsy quality-of-care measures. Neurol. Clin. Prac..

[37] Wicks P., Vaughan T.E., Massagli M.P. (2012). The multiple sclerosis rating scale, revised (Msrs-R): Development, refinement, and psychometric validation using an online community. Health Qual. Life Outcomes.

[38] Little M., Wicks P., Vaughan T., Pentland A. (2013). Quantifying short-term dynamics of parkinson’s disease using self-reported symptom data from an internet social network. J. Med. Int. Res..

[39] Swan M. (2012). Crowdsourced health research studies: An important emerging complement to clinical trials in the public health research ecosystem. J. Med. Int. Res..

[40] Inspire. http://www.inspire.com/.

[41] Trialx. http://trialx.com/.

[42] Army of women. http://www.armyofwomen.org/.

[43] Vayena E., Tasioulas J. (2013). Adapting standards: Ethical oversight of participant-led health research. PLoS Med..

[44] Vayena E., Mastroianni A., Kahn J. (2013). Caught in the web: Informed consent for online health research. Sci. Transl. Med..

[45] Foddy B. The Ethics of Gamification: Little Rewards for Everything. http://blog.practicalethics.ox.ac.uk/2011/02/the-ethics-of-gamification-little-rewards-for-everything/.

[46] Gutmann A., Wagner J.W. (2013). Found your DNA on the web: Reconciling privacy and progress. Hastings Center Rep..

[47] Roche P., Glantz L.H., Annas G.J. (1996). The genetic privacy act: A proposal for national legislation. Am. Bar Assoc..

[48] Rothstein M.A. (2010). Is deidentification sufficient to protect health privacy in research?. Am. J. Bioeth..

